# Distinguishing neuronal from astrocytic subcellular microstructures using *in vivo* Double Diffusion Encoded ^1^H MRS at 21.1 T

**DOI:** 10.1371/journal.pone.0185232

**Published:** 2017-10-02

**Authors:** Noam Shemesh, Jens T. Rosenberg, Jean-Nicolas Dumez, Samuel C. Grant, Lucio Frydman

**Affiliations:** 1 Champalimaud Neuroscience Programme, Champalimaud Centre for the Unknown, Lisbon, Portugal; 2 The National High Magnetic Field Laboratory, Florida State University, Tallahassee, Florida, United States of America; 3 Institut de Chimie des Substances Naturelles, CNRS UPR2301, Gif-sur-Yvette, France; 4 Florida State University, Chemical & Biomedical Engineering, Tallahassee, Florida, United States of America; 5 Department of Chemical Physics, Weizmann Institute of Science, Rehovot, Israel; National Research Council of Italy, ITALY

## Abstract

Measuring cellular microstructures non-invasively and achieving specificity towards a cell-type population within an interrogated *in vivo* tissue, remains an outstanding challenge in brain research. Magnetic Resonance Spectroscopy (MRS) provides an opportunity to achieve cellular specificity via the spectral resolution of metabolites such as N-Acetylaspartate (NAA) and myo-Inositol (mI), which are considered neuronal and astrocytic markers, respectively. Yet the information typically obtained with MRS describes metabolic concentrations, diffusion coefficients or relaxation rates rather than microstructures. Understanding how these metabolites are compartmentalized is a challenging but important goal, which so far has been mainly addressed using diffusion models. Here, we present direct *in vivo* evidence for the confinement of NAA and mI within sub-cellular components, namely, the randomly oriented process of neurons and astrocytes, respectively. Our approach applied Relaxation Enhanced MRS at ultrahigh (21.1 T) field, and used its high ^1^H sensitivity to measure restricted diffusion correlations for NAA and mI using a Double Diffusion Encoding (DDE) filter. While very low macroscopic anisotropy was revealed by spatially localized Diffusion Tensor Spectroscopy, DDE displayed characteristic amplitude modulations reporting on confinements in otherwise randomly oriented anisotropic microstructures for both metabolites. This implies that for the chosen set of parameters, the DDE measurements had a biased sensitivity towards NAA and mI sited in the more confined environments of neurites and astrocytic branches, than in the cell somata. These measurements thus provide intrinsic diffusivities and compartment diameters, and revealed subcellular neuronal and astrocytic morphologies in normal *in vivo* rat brains. The relevance of these measurements towards human applications—which could in turn help understand CNS plasticity as well as diagnose brain diseases—is discussed.

## Introduction

Neurons and astrocytes comprise the brain’s two major cell populations, and are critical for realizing and supporting this organ’s complex functions [1]. Many neurodegenerative diseases are characterized by changes in the morphologies of these cells and/or their intracellular substructures, which can become aberrant early in disease progression [2,3]. The detection of cellular-specific microscopic features—and, in particular, the ability to distinguish different subcellular properties of specific cells—could thus serve as an important way of diagnosing disease, as well as understanding the maturation of brain and course of neurodegeneration. Such processes can be monitored either *post-mortem* or *in vivo*, but only invasively and with a limited degree of cellular specificity using the injection of contrast agents [4–6]. By harnessing specific chemical signatures, Magnetic Resonance Spectroscopy (MRS) techniques detecting weak but cell-specific metabolic signals could open a unique window to performing similar observations in a non-invasive, *in vivo* fashion [7–11]. Whereas chemical shift-based measurements are usually employed to estimate metabolite concentrations [12–15], microstructural features have been shown amenable to elucidation via diffusion-weighted MRS [7,8,10,16–20]. Single Diffusion Encoding (SDE) filters [21] were initially used for this purpose; however, the resulting Diffusion Tensor Spectroscopy (DTS) measurements will often lack the ability to reflect microscopic anisotropies due to the highly heterogeneous, randomly-ordered nature of most brain-relevant voxels used for spectroscopy. Double Diffusion Encoding (DDE) MR [22,23], by contrast, is emerging as a promising metric for characterizing highly heterogeneous, disordered biological structures [23–29]. In the context of *in vivo* MRI measurements, DDE studies have included experiments in rats [30], as well as human studies using clinical scanners [25,31–33,33–36]. DDE experiments operate by creating diffusion correlations between two diffusion encoding events along various gradient-imposed orientations [37,38]. Thanks to this dual encoding mode, the ensuing contrast decouples the sought-after microscopic anisotropy (μA) from the overall orientation dispersion of the studied objects at long mixing times [22,29,34,39,40]. As such, the experiment can report on μA that characterizes pore morphology—representing its local eccentricity—regardless of the macroscopic orientation dispersion properties of the object in question [26,27,34]. Measurements of μA are not necessarily confined to the DDE class of experiments: the so-called qMAS experiment can reveal μA by rotation of the q-vector along the magic angle [41,42], while other recent approaches borrow concepts from solid-state NMR to resolve μA from 2-dimensional correlations between isotropic and anisotropic encoding elements of the diffusion weighting tensor (de Almeida Martins and Topgaard 2016).

In DDE experiments, μA is measured by varying the angle ψ between two diffusion-encoding gradients separated by long mixing times [22,39]. At long mixing times, a cos(2ψ)-like amplitude modulation can emerge, whose depth at a given q-value (where ***q*** = *(2π)*^*-1*^*γδ****G***, *γ* is the gyromagnetic ratio, *δ* is the gradient duration, and **G** the gradient vector) will report on μA [26,36,39]. When applied to the water resonance, DDE experiments provides insight into the local morphology of heterogeneous systems [27,29,30,37]. Cellular specificity, however, cannot be attained via such DDE measurements due to ubiquity of water throughout cells. We have recently shown that DDE contrast can be imparted onto metabolic signals *in vivo* and observed with good sensitivity, using a methodology dubbed “Relaxation Enhanced” MRS (RE-MRS) [43]. RE-MRS exhibits particular advantages at ultrahigh fields, as the use of spectrally-selective radiofrequency (RF) pulses targets (by excitation and/or refocusing) only resonances of interest, while avoiding water excitation. This provides spectra with excellent line shapes, as well as potential sensitivity enhancing effects arising from transfers from an intact magnetization reservoir [44–46]. RE-MRS experiments have enabled robust *in vivo* measurements of relaxation properties for non-exchangeable protons [47], as well as measurements on exchangeable resonances [43] such as NAD^+^ nucleotide signals [48].

Out of the spectral choices available for restriction length-scales measurements, N-Acetylaspartate (NAA) and myo-Inositol (mI) stand out as the most advantageous MRS reporters for characterizing specific cellular populations’ microstructure. Indeed, these metabolites are largely confined to distinct cellular spaces [49,50]: NAA is found almost exclusively in neurons, both in the cell body as well as in neurites and axons [9,51], while mI is predominantly—though likely not exclusively—found in the astrocytic cell bodies and branches [49,50]. Le Belle *et al* have shown that other observable metabolites like total cholines show predominance in astrocytes [52]; however, it is likely that up to 25% of the choline resonance also emerges from neuronal cell membranes. NAA and mI, therefore, can potentially report on various properties of a cellular population with high specificity. *In vitro* studies have used the q-space profiles of NAA as a marker for neuronal signals [53], while more recently Ronen *et al* [54] and Najac *et al* [55] have performed diffusion weighted spectroscopy (DWS) measurements in humans at 7 T using NAA as neuronal and tCho/tCre as astrocytic-based markers. Others were able to measure apparent diffusion coefficients (ADCs) for these metabolites in normal [56,57] and pathological [58,59] conditions. In most of these studies the elusive mI resonance is rarely reported, and its more specific environmental information not exploited. Only a very recent study attempted to decipher the local environment in neurons and astrocytes by probing multiple metabolites—including NAA and mI—at multiple diffusion times, and fitting the ensuing signal decay characteristics to an elaborate model of (intra)-cellular micro-architecture [60]. These fits suggested that many metabolites were mainly confined to randomly oriented sub-cellular anisotropic structures, such as neuronal dendrites or axons and astrocytic branches [60].

Here we sought to exploit ^1^H RE-MRS on NAA and mI at ultrahigh fields, to investigate whether their DDE signatures can report on cellular-specific micro-architectural features of *in vivo* brains. Under the experimental conditions available in our 21.1T system, conventional spectroscopic variants like STEAM failed to achieve the high fidelity that such DDE experiments require—probably as a result of shimming and RF limitations that prevented us form harnessing outer volume suppression or efficient water suppression during the DDE mixing time. By contrast, our novel RE-MRS variant involving a Carr-Purcell-Meiboom-Gill (CPMG) train during the DDE filter provided excellent quality spectra while overcoming putative internal susceptibility-driven gradients arising at ultrahigh fields. With these provisions, we find that sufficient sensitivity could be achieved to endow these cell-specific *in vivo* DDE experiments with sufficient signal-to-noise ratio (SNR) to enable simultaneous detection of mI and NAA signals as cellular-specific reporters. The DDE amplitude modulations observed for these two targeted metabolites reflected microscopic anisotropies in NAA’s and mI’s diffusion, which we ascribe to the confinements felt in randomly oriented neurites and astrocytes respectively, thereby providing direct experimental evidence to Palombo et al’s model [60].

## Materials and methods

All experiments were preapproved by the Florida State University Animal Care and Use Committee. Every effort was taken to minimize animal suffering. The experiments took place at the US National High Magnetic Field Laboratory, under the direction of a veterinarian who is certified as a specialist in laboratory animal medicine by the American College of Laboratory Animal Medicine (ACLAM). All animal procedures were carried in accordance with the guidelines for animal experimentation from the ethical committee of the Florida State University Animal Care and Use Committee. FSU is registered as a research facility with the United States Department of Agriculture (USDA Registration #58-R-0001) and has an Animal Welfare Assurance number (#A3854-01) on file with the US Public Health Service. All animal procedures were undertaken according to these regulatory bodies and AAALAC guidelines. The experiments took place at the US National High Magnetic Field Laboratory’s 21.1 T MRI/NMR (900 MHz ^1^H frequency [61,62], equipped with a Bruker Avance III console, a RRI Inc. gradient system capable of producing up to 60 G/cm in all dimensions, and a customized quadrature surface coil probe [63]. Naïve Sprague Dawley rats were purchased from Envigo Corp. (Tampa, FL, USA) and delivered one week prior to surgery for acclimatization to the new environment. All animals were housed individually in a 12 hr night/12 hr daylight cycle with water and food (Rodent chow, Purina, Ontario Canada) available *ad libitum*. Healthy Sprague-Dawley rats (N = 6, one animal was excluded from analysis due to spectroscopic artefacts) weighing between 200–250 gr were randomly chosen from the cohort of available animals. Prior to their *in vivo* imaging all animals were anesthetized with 5% isoflurane (Henry Schine Inc Melville, NY) and then lowered to 2–3% once the animal was sedated. The animal eyes were prepared with an eye ointment (Artificial Tears Ointment, Revival Animal Health, Inc, Orange City, IA) to protect the cornea from drying while in the magnet. The animal was carefully placed in an animal cradle and with its front teeth on a bite bar, where Isoflurane and O_2_ were continuously administrated through. The animal was then carefully pulled into the coil over a pneumatic pillow, and the isoflurane was adjusted to maintain a respiratory rate between 30–40 breaths/min (SA Instruments Inc. Stony Brook, NY). All imaging, including scout images and MRS experiments, took no longer than 2 hours. Euthanasia was performed in guidance with the 2007 American Veterinary Medical Association (AVMA) Guidelines on Euthanasia. After the last MRS session animals were anesthetized with isoflurane and exposed to CO_2_ for a fast and pain free euthanasia. Once respiratory failure and cardiac arrest had occurred, a cervical dislocation was performed.

**Fig 1:**
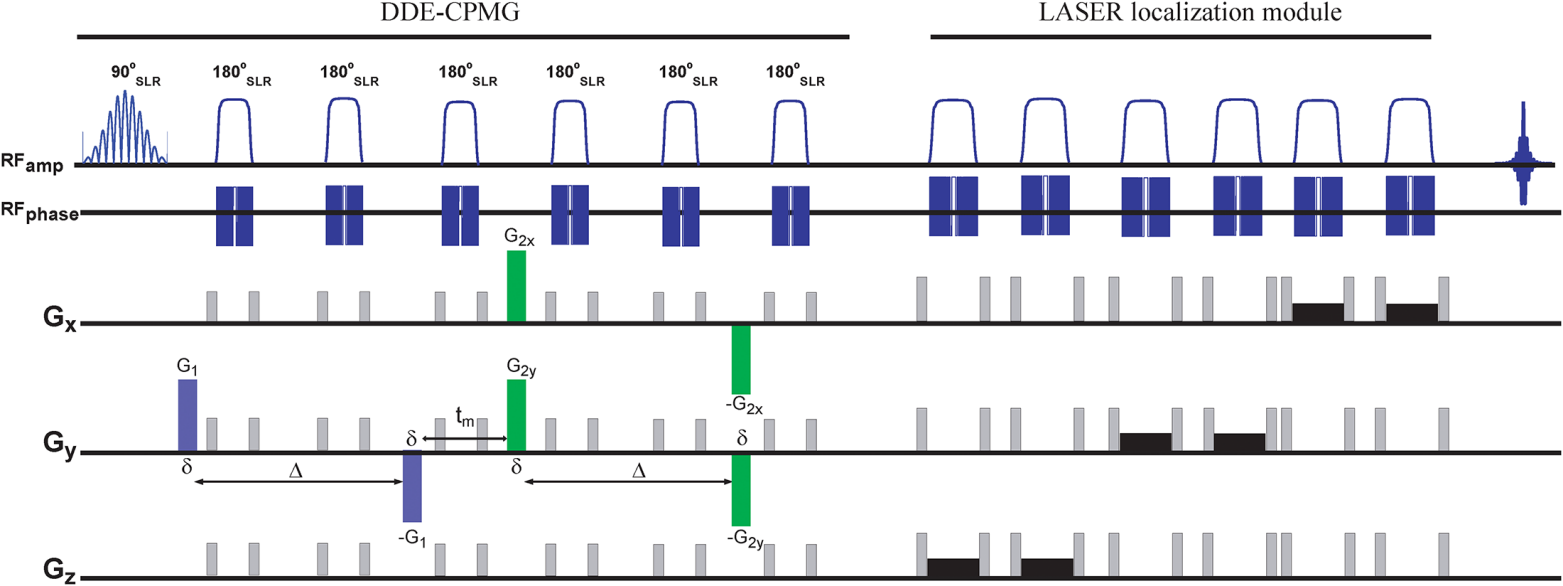
DDE RE-MRS sequence incorporating LASER localization and CPMG modules that mitigate the cross-terms arising from internal gradients. The 8 ms spectrally-selective excitation pulse comprised two narrow bands targeting the NAA singlet resonance at 2.02 ppm and the mI multiplets at 3.51 and 3.61 ppm. This DDE-CPMG module was followed by refocusing and adiabatic LASER pulses. All spectrally-selective pulses were designed using the SLR algorithm (Pauli et al, 1991). The correlated DDE gradients are shown in blue and green. Crusher gradients are shown in grey and slice-selective gradients in black. Acq = acquisition.

**Fig 2:**
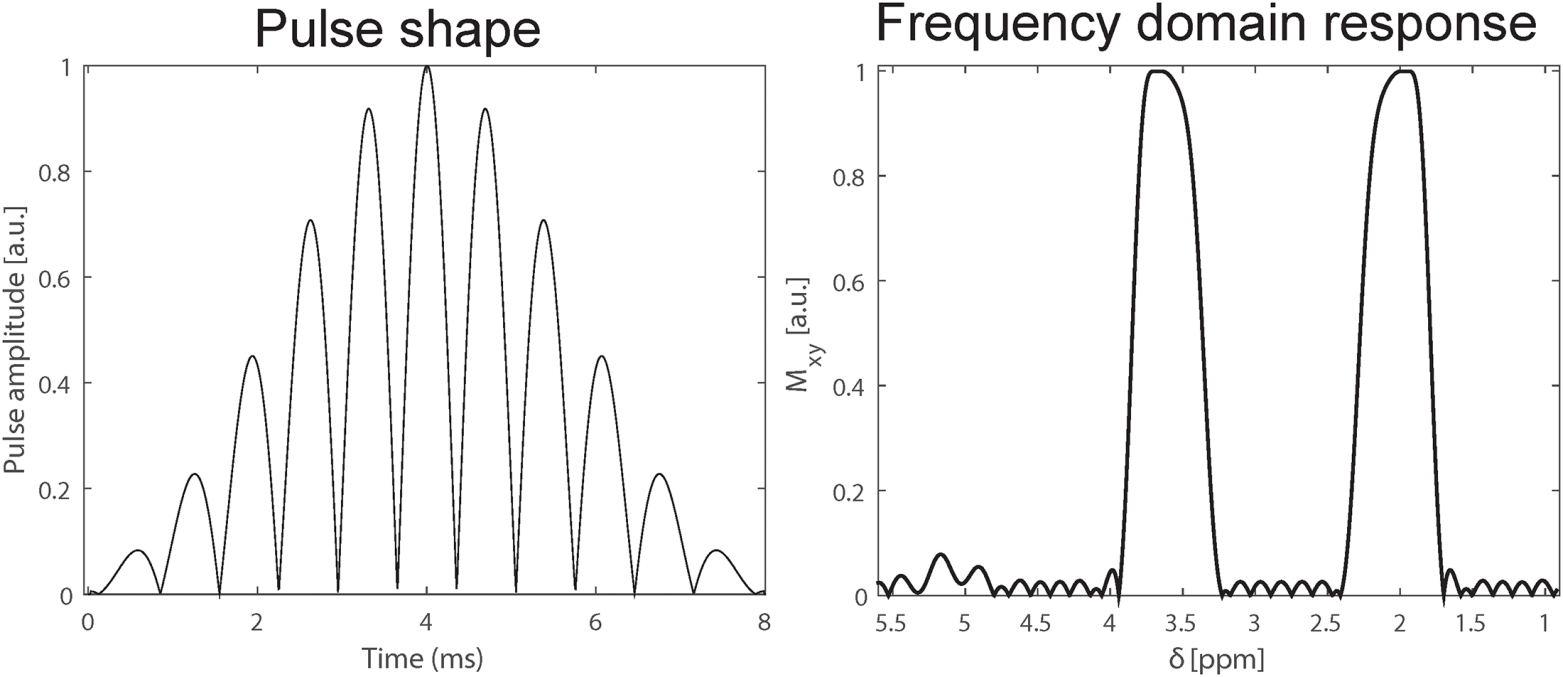
RF pulse shape used to selectively address mI and NAA (left) and its frequency spectrum (right). The bandwidths of the 8 ms pulse are sufficiently narrow to efficiently convert all magnetization aligned initially along M_z_ into M_xy_ magnetization within the desired bands, but not elsewhere. This results in a cellular-specific mode of excitation.

In general, the RE-MRS approach (Fig 1) entails spectrally-selective excitation and refocusing RF pulses, which simplify the spectra, avoid distortions and enhance sensitivity. In this study, 8 ms pulses were used (Fig 2) possessing two excitation bands incorporating the NAA singlet resonance at 2.02 ppm and mI multiplet resonances at ~3.51 and ~3.61 ppm. Note that compared to our previous studies (Shemesh et al 2014), the frequency profile of the pulses contained ripples of <1% around the water resonance; while this allowed use of shorter pulses it did not mitigate putative relaxation enhancement effects. Despite this residual ≈1% ripple no water suppression was needed or applied, as the elicited water signal was dephased via either T2 relaxation or by the crusher gradients applied. Spatial localization was achieved prior to the spectral acquisition via a LASER module, encompassing three pairs of 5 ms spatially-selective refocusing pulses [64]. This localization was verified by imaging the voxel directly via a fast low-resolution T_2_-weighted sequence that incorporates a LASER module. Shimming was performed manually using up to 4^th^ order corrections, with ~30 Hz line widths typically observed on the voxel of interest. Line broadening factors equal to the line widths were applied upon processing the spectra, which were all analysed in magnitude mode to avoid phasing complications and/or the need to do scan-by-scan phase correction (Ligneul and Valette, 2017). All this led to somewhat broader lines, of ca. 0.07 ppm FWHHs.

**Fig 3:**
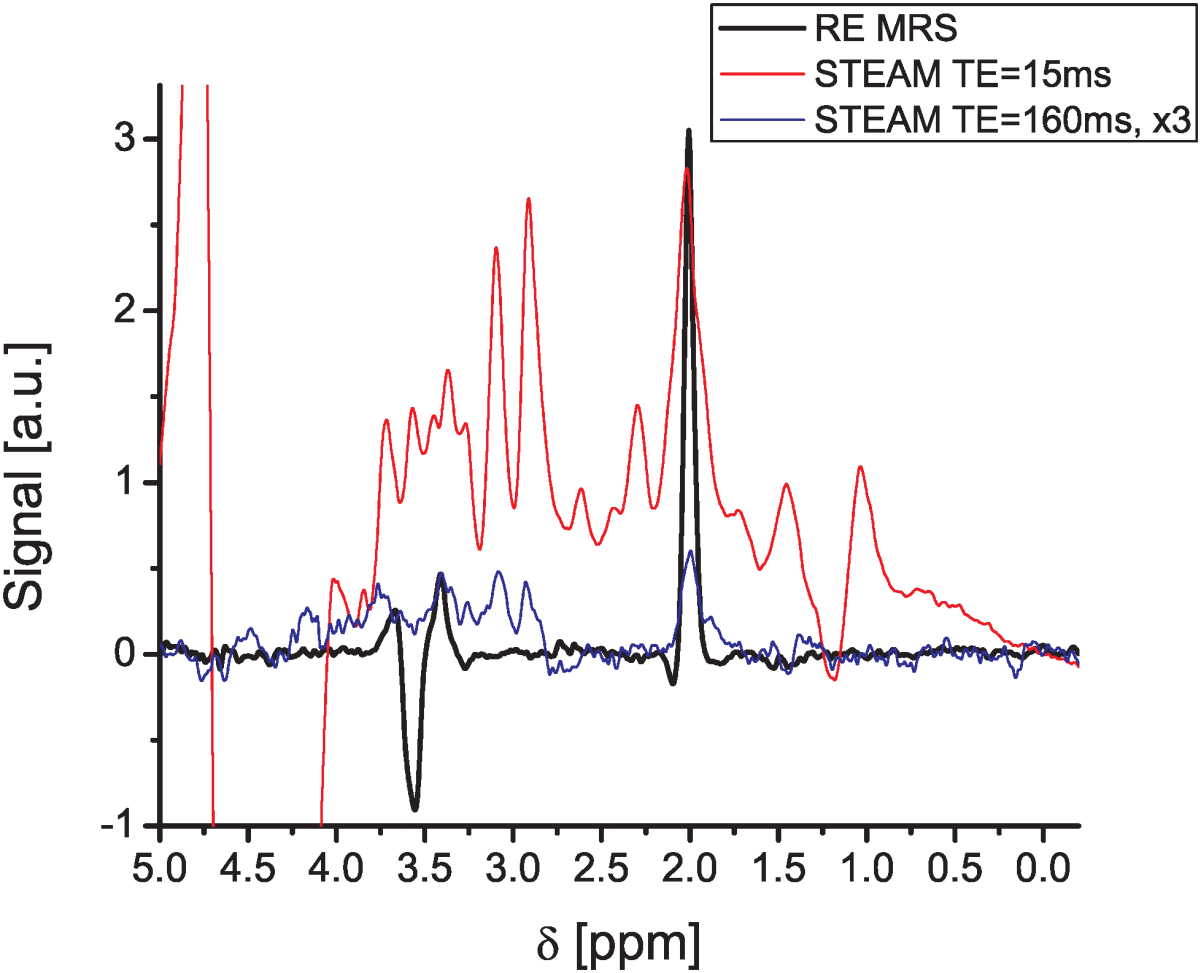
Direct comparison between RE MRS and STEAM spectra collected for different echo times, performed at 21.1T using identical RF and spectral width components. All spectra are shown in magnitude mode; notice the strong water-induced distortion in the mI region arising from the STEAM sequence at short TE, as well as the small SNR for the targeted metabolites for STEAM at TE = 160ms (a similar effective TE as used for the RE MRS experiment), where it had to be multiplied by 3 to be visible on this graph. Experiments were acquired under identical conditions in terms of voxel sizes (100 μL) and positions, over 10 min (256 scans) acquisition times. Similar results were observed over N = 6 independent tests. We ascribe the poor water suppression performance of the STEAM sequence to limitations in the sensitivity-optimized surface coil 900 MHz setup used, to accurately impart the large-angle nutations needed over the relevant voxel.

The importance of relying on the RE MRS sequence for these experiments was investigated by comparing its non-diffusion-weighted NAA/mI spectra, against counterparts arising from the scanner’s built-in STEAM sequence after optimization and incorporation of VAPOR water suppression [65]. Acquisition parameters were kept identical inasmuch as possible between both sequences (voxel size, voxel location, repetition time, etc) except for the TE, which for STEAM was also explored for potentially shorter values. Fig 3 shows representative results of these comparisons; clearly, the STEAM MRS executed for shorter TEs shows better sensitivity but also a much stronger residual water resonance than the longer TE counterpart—whose SNR would be simply too low for incorporating diffusion-weighting. Our STEAM’s poor water suppression performance could likely be improved using outer-volume-suppression and further water suppression modules during the TE; however, RF limitations prevented us from implementing the latter, and so this was not implemented in this study. The RE-MRS trace possesses a flatter baseline than the short-TE STEAM counterpart at the mI resonance position (an essential feature for quantifying diffusion-driven modulations in these long acquisitions) but also a superior SNR: ≈1.5x higher for the mI peak and ≈4x better for NAA. PRESS experiments potentially characterized by higher SNRs than STEAM were also assayed, but in the 21.1 T-optimized quadrature-surface coil probe employed these required a strong RF power for the spins’ refocusing and localization that was unavailable; PRESS experiments therefore failed to provide meaningful results.

To reliably incorporate a long DDE filter, six refocusing pulses were incorporated into the RE MRS sequence (Fig 1). In effect these pulses will modulate the internal (background) gradient spectrum away from the gradient spectrum applied by the DDE, thereby mitigating cross-terms between these two. We chose our experimental DDE parameters using the Microstructure Imaging Sequence Simulation Toolbox (MISST) [66–68], with which we simulated the conditions whereby the DDE sequence would be sensitive to randomly oriented, anisotropic compartments. These simulations resulted in: δ = 4.5 ms, Δ = 64 ms, t_m_ = 25 ms **|G**_**1**_**| = |G**_**2**_**|** = 24 G/cm. Other parameters were TR/TE = 2500/167 ms and NA = 160. Angular DDE was carried out by changing, in the X-Y plane, the relative angle between **G**_**1**_ and **G**_**2**_ in 45° increments between 0 and 360 degrees. Additional planes (X-Z and Y-Z) were probed, but no significant differences among their results were found and DDE data are hence circumscribed to X-Y rotations. Rather than relying on specific orthogonal measurements, full fits of the rotational plots were implemented in order to better assess the DDE responses. These DDE signals were examined closely for an optimum model selection; e.g., finite vs. infinite cylinders. Given the large amplitude modulations observed under the specific parameters above, and in accordance with the results given in [60], the infinite, randomly oriented cylinder model was chosen, where the two critical parameters are the cylinder diameter and intrinsic diffusivity. The ensuing DDE amplitude modulations *E(ψ) = S(ψ)/S(q = 0)*, where *S(ψ)* describes the signal as function of the relative angle between the gradients and *E(ψ)* is the relaxation-normalized angular variation, were then compared against a library of signals calculated in MISST [66–68], which include a range of parametric signals. In particular, we used MISST to simulate the signals arising from infinite cylinders; this particular model was chosen after initial tests with finite cylinder models were seen to provide similar results. Diameters were varied between 0.1–5.0 μm, and intrinsic diffusivities between 0.13 and 0.65 μm^2^/ms. Least square residuals were then computed between the experimental data and simulated dataset, with the minimum residual chosen as the best fit. To compare the statistical significance of differences between NAA and mI, the extracted diameters and intrinsic diffusivities were subject to a paired Student’s t-test, with p = 0.05 thresholding the validity of the null hypothesis. Simulations for gradient dephasing spectra were performed numerically using in-house written code, which performed a numerical evaluation of each gradient waveform as described in Equation 16 of Zheng *et al* [69].

In complement to the DDE measurements, diffusion tensor spectroscopy (DTS) measures of macroscopic fractional anisotropy (FA) were performed on a separate set of N = 4 rats, focusing on an identical voxel location as the DDE. The experiments were carried out using the same RE-MRS pulse sequence described above, scanning **G**_**1**_ in 6 directions to measure while setting |**G**_**2**_| = 0, thereby effectively achieving a SDE encoding. The tensor elements were extracted by a nonlinear fit of the data in Matlab^®^ (Mathworks Inc., Nantick MA), and eigenvalues and eigenvectors computed following diagonalization.

All of this paper’s DDE data are provided as Supporting Information (S1 File). This DDE MRS data is sorted according to animal and gradient strength/direction, and stored in folders with the format animalX\3Y –where X was the X^th^ animal studied and Y was the Y^th^ experiment performed in that animal. For each animal’s folder, the first experiment corresponds to G = 0 and the next experiments are ordered according to psi = 0:45:360. I.e., in the series 30–39, 30 is the experiment with G = 0 and the variable-angles correspond to experiments 31–39. If an experiment number has the suffix 'o' (e.g., 35o), it means that an artifact was detected in 35 and that the experiment was repeated. These individual DDE data sets for the various animals scanned, can be found in marked folders of the Supporting Information (S1 File) as individual Bruker-formatted files.

## Results

**Fig 4:**
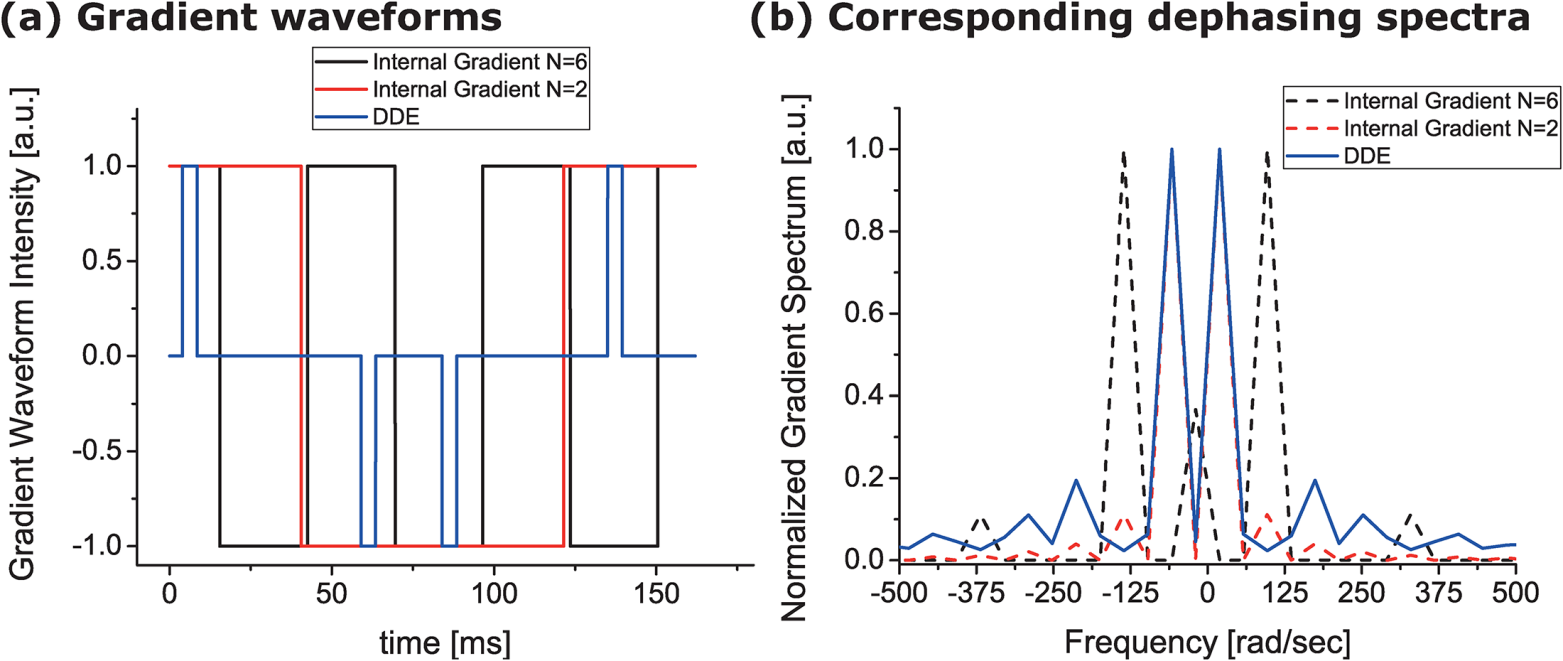
Analyzing the coupling between the DDE gradient waveform and potentially deleterious internal gradients modulations brought about by RF spin echoes. (a) Temporal evolution of the gradient waveform for the DDE filter (blue) and for the internal gradient waveform modulated by N = 2 CPMG (red) and N = 6 CPMG modulation. These waveforms account for the effects of refocusing pulses. (b) Corresponding dephasing spectra for the three waveforms. Notice that, for N = 6 CPMG, the internal gradient and the DDE spectra are minimally overlapping, suggesting a strong suppression of their cross-term. By contrast, there is significant overlap between the N = 2 CPMG and DDE spectra, explaining the potential vulnerability of the latter sequence towards susceptibility-induced cross-terms which may corrupt the desired information.

The DDE RE-MRS pulse sequence used in this study (Fig 1) was tailored to two ~600 Hz wide frequency-selective bands at 2.02 and 3.58 ppm, whereby only NAA, mI and (to a lesser extent) taurine, were excited. Taurine’s excitation, however, should not interfere with the mI measurements, as taurine is well resolved in the spectrum. The water resonance around 4.7 ppm was excited to well below 0.1% of its maximum (Fig 2), leaving most of the water spins unperturbed. Following this excitation, a DDE module was applied, incorporating a CPMG spin-echo sequence [70] based on selective SLR pulses. This CPMG module was required to mitigate potential interactions between the DDE gradients and susceptibility-induced gradients—internal fields that at ultrahigh fields could not be neglected and which could distort the DDE curves if unaddressed [69,71]. To better appreciate this need, gradient dephasing spectra were calculated and used to analyze the cross-terms between a given diffusion-sensitizing waveform and the internal gradient waveform (see Zheng *et al* for an extensive review on gradient dephasing spectra and internal gradients [69]). Fig 4 summarizes these results by showing the relevant gradient waveforms associated to our past and present DDE experiments (Fig 4a), as well as the spectral distributions associated with each of these modulations (Fig 4b) assuming a susceptibility-imposed background gradient that is temporally independent but is modulated by the RF inversion pulses. A strong overlap between the RF-modulated spectral density and DDE waveform can bring about a fast, undesirable dephasing that masks the information being sought. As can be appreciated from Fig 4b, the DDE spectrum (blue) significantly overlaps with the internal gradient dephasing spectrum for the previously used two-pulse CPMG sequence (red), but exhibits a much weaker overlap with the new N = 6 pulses CPMG sequence (black) employed in this study. The significantly reduced overlap reduces the cross-term—by a factor of ~30 upon comparing N = 2 and N = 6 experiments. This in turn explains the robustness of the latter sequence towards potential susceptibility-driven cross-term artifacts.

**Fig 5:**
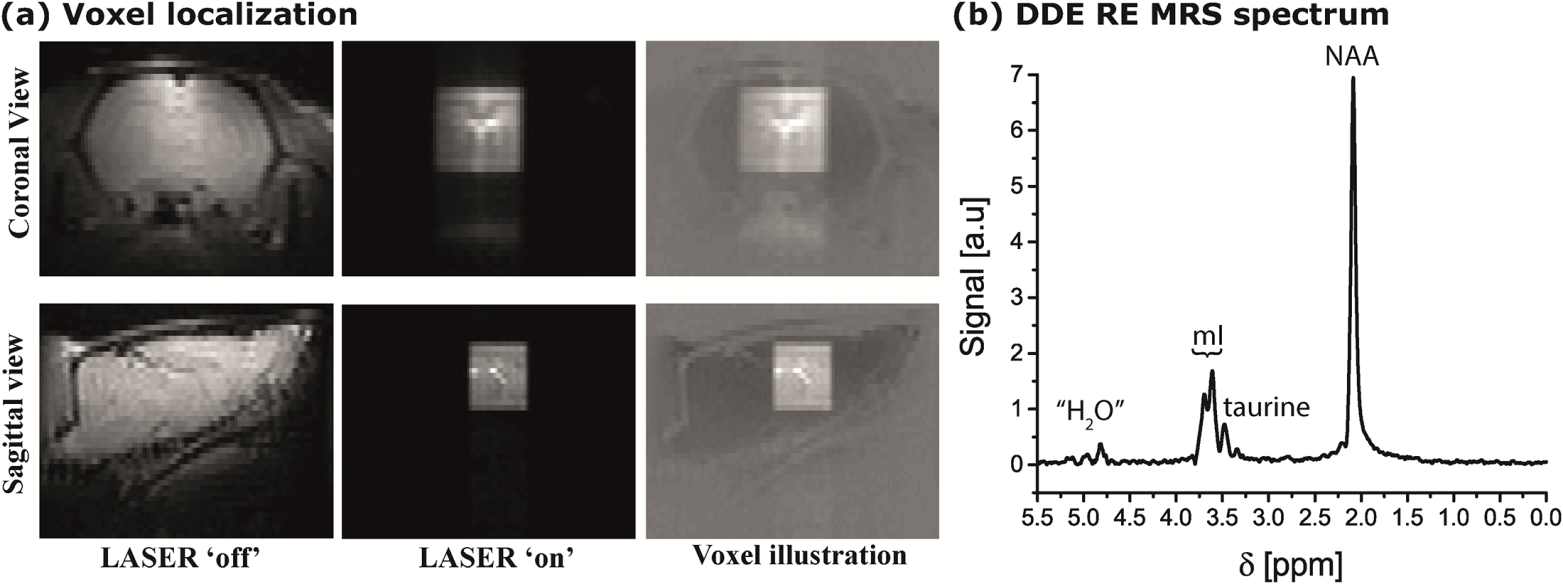
Examples of the voxel localization and of spectra obtained in representative DDE RE-MRS *in vivo* experiments. (a) Coronal (upper panel) and sagittal (lower panels) views of the brain via a low-resolution T_2_-weighted water-based imaging experiment, applied without (left most images) and with (middle panels) a LASER localization module identical to the one that was subsequently applied in DDE RE-MRS. A clearer view of the voxel is presented in the right-hand panel of (a), where the voxel image is overlaid on a darkened image of the brain. (b) Representative spectrum arising from the 125 μL voxel shown in (a), collected in ~6.5 min and showing the targeted resonances, together with small residuals from water and from a nearby taurine resonance.

With this increased robustness towards internal gradients secured, an accurate voxel localization was achieved by LASER [64]; this is illustrated in Fig 5a for both coronal and sagittal views of an *in vivo* rat brain at 21.1 T. The (5 mm)^3^ targeted voxel is robustly localized along all three dimensions. A representative spectrum arising from this localized region is shown in Fig 5b. Clearly, the NAA and mI peaks of interest are excited selectively (noting the minor taurine signal evident within the mI bandwidth) and observed with excellent fidelity, despite the usual complications of resolving mI with adequate sensitivity at either using STEAM with water suppression (Fig 3) or at lower fields [72]. With diffusion encoding gradients set to zero, typical SNRs observed were 400 and 85 for NAA and mI, respectively, after averaging 160 scans (total time of ~6.5 minutes per trace). Note that the selective pulses are the dominant source of spectral specificity in these experiments, obviating the need for water suppression and its sensitivity/spectral-distortion penalties; at the same time, this RE-MRS approach provides sufficient sensitivity to measure the cellular-specific microstructures.

**Fig 6:**
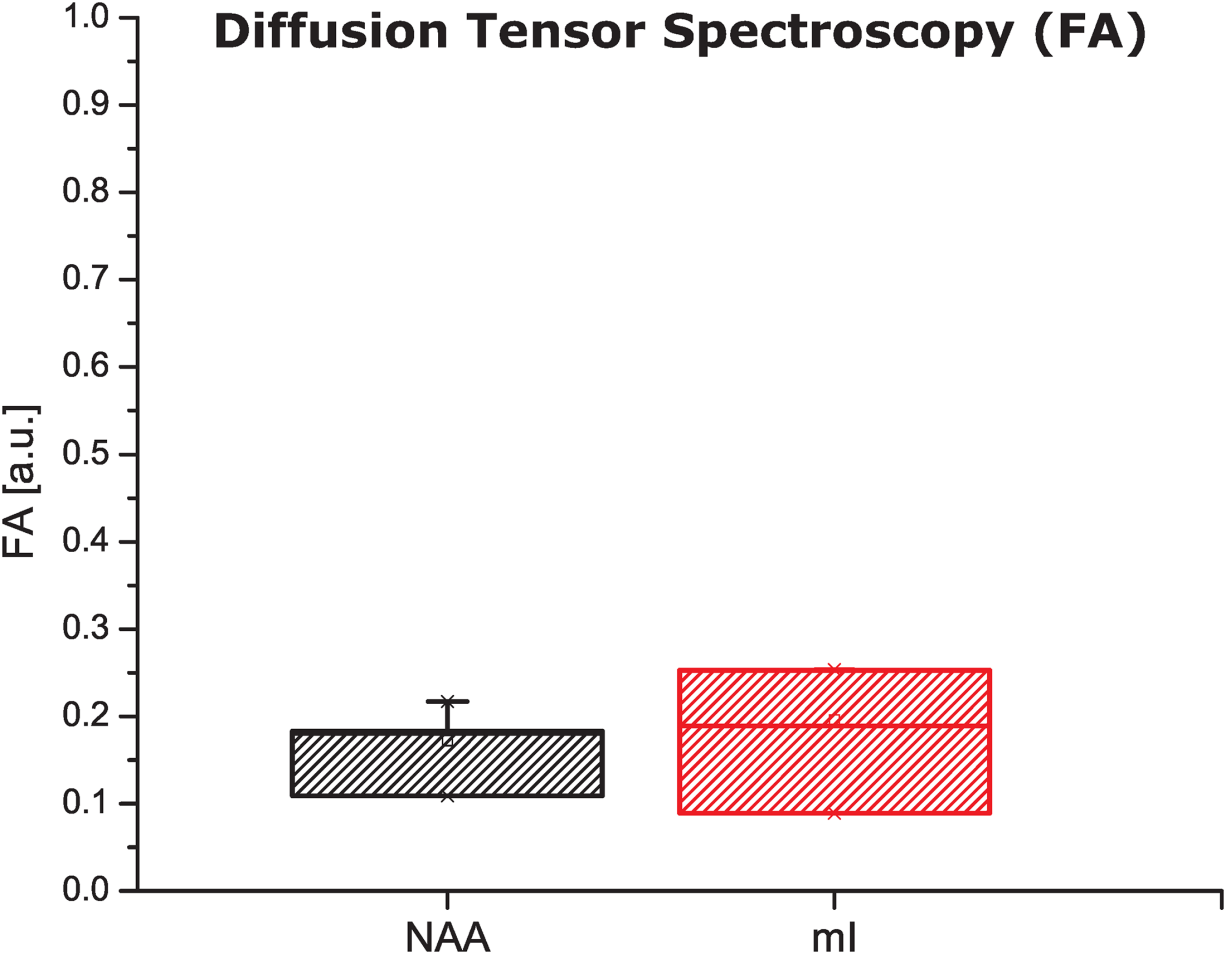
Fractional anisotropies (FAs) derived from diffusion tensor spectroscopy analyses at 21.1T. As a result of the large voxel targeted in the rat brain, the averaging of diffusion directors over many orientations results in a negligible macroscopic anisotropy for both metabolites. The lower variability measured for NAA predominantly reflects the higher effective SNR for this singlet resonance (compared to the multiplet mI). Results reflect measurements on *n* = 4 animals.

Several MRS studies have been able to impart diffusion-based contrasts on *in vivo* metabolic signals, most of them aiming to obtain information on the diffusion properties of specific compartments given some metabolites’ specificity [10,16–19,54–59,73–77]. Other *ex vivo* studies performed q-space measurements yielding the restricting length scale for NAA, which was compared with the water-based diffusion [53]. Direct evidence for μA experienced by several metabolites in brain was recently reported [43], substantiating (among others metabolites) NAA’s restricted diffusion within randomly oriented but locally anisotropic neuronal compartments. Palombo *et al*’s recent modeling work is also consistent with these findings [60]. In order to explore whether under the spatially-localized conditions used in this work the metabolites would exhibit a sizable global diffusion anisotropy, DTS measurements were performed using a single-pulsed gradient SDE sequence. Fig 6 shows bar plot of the macroscopic fractional anisotropy thus measured for both metabolites, on similar voxel sizes and locations as illustrated in Fig 5. Very low FAs are evidenced for both metabolites, with average values for NAA and mI of *FA*_*NAA*_ = 0.17 ± 0.05, *FA*_*ML*_ = 0.19 ± 0.08 respectively. These very low FA values reflect the fact that for the relatively large brain voxel sizes that we chose to analyze, the randomly oriented model is a good approximation for this system: on the voxel scale chosen, pore directors would be approximately powder averaged and anisotropic information lost.

**Fig 7:**
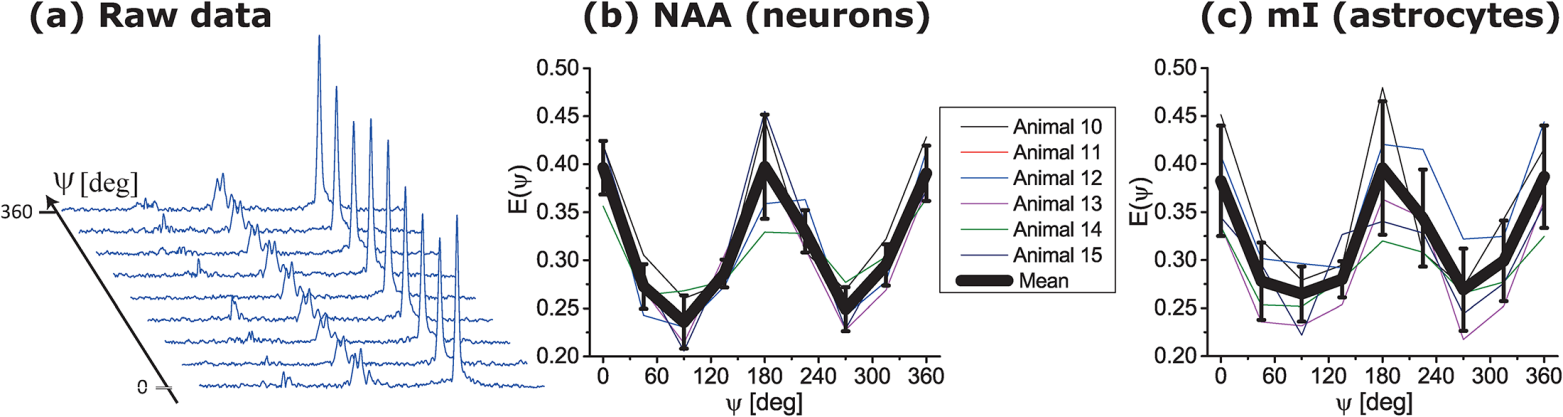
Probing micro-architectural features of neurons and astrocytes by *in vivo* DDE RE-MRS on the cell-specific metabolites NAA and mI. (a) Illustrative spectral set collected at 21.1 T in 160 scans (~6.5 min) as a function of the angle ψ between the diffusion-sensitizing gradients. Further details on these experiments are given in Methods. (b,c) Peak oscillations observed for the two metabolites in a series of experiments collected from *N = 6* animals, together with the mean oscillations fitted from these experiments.

Given the nearly isotropic metabolic diffusion revealed by these SDE measurements, we proceeded to assess what kind of microscopic anisotropy mI experiences compared to NAA, when assessed by DDE. The major DDE “fingerprint” for this μA will be the amplitude modulation of *E(ψ)*, whose depth is dependent on the variance of the diffusion tensor eigenvalues of the principal axis within a compartmental pore, which in turn can reflect the eccentricity of the compartment. *In vivo* DDE RE-MRS experiments were conducted simultaneously on a number of rat brains. The raw data (Fig 7a) revealed characteristic signal amplitude modulations for both metabolites, evidencing that mI also undergoes restricted diffusion within anisotropic, albeit randomly oriented, astrocytic environments. Fig 7b and 7c show the NAA and mI signals, respectively, for several animals studied, along with the mean amplitude modulation for each metabolite plotted in thick black lines. The signal at ψ = π/2 is different from the signals at ψ = 0 for both metabolites in a statistically significant manner (p<0.005), demonstrating the restricted diffusion experienced by both NAA and mI. These amplitude modulations strongly suggest that the microstructures where NAA diffuses are highly anisotropic at a local level, in agreement with several recent, model-based studies [17,60]. Notice as well the absence of an initial phase shift in these DDE rotation plots, which would be indicative of marginal bulk alignment (Shemesh et al, 2012).

**Fig 8:**
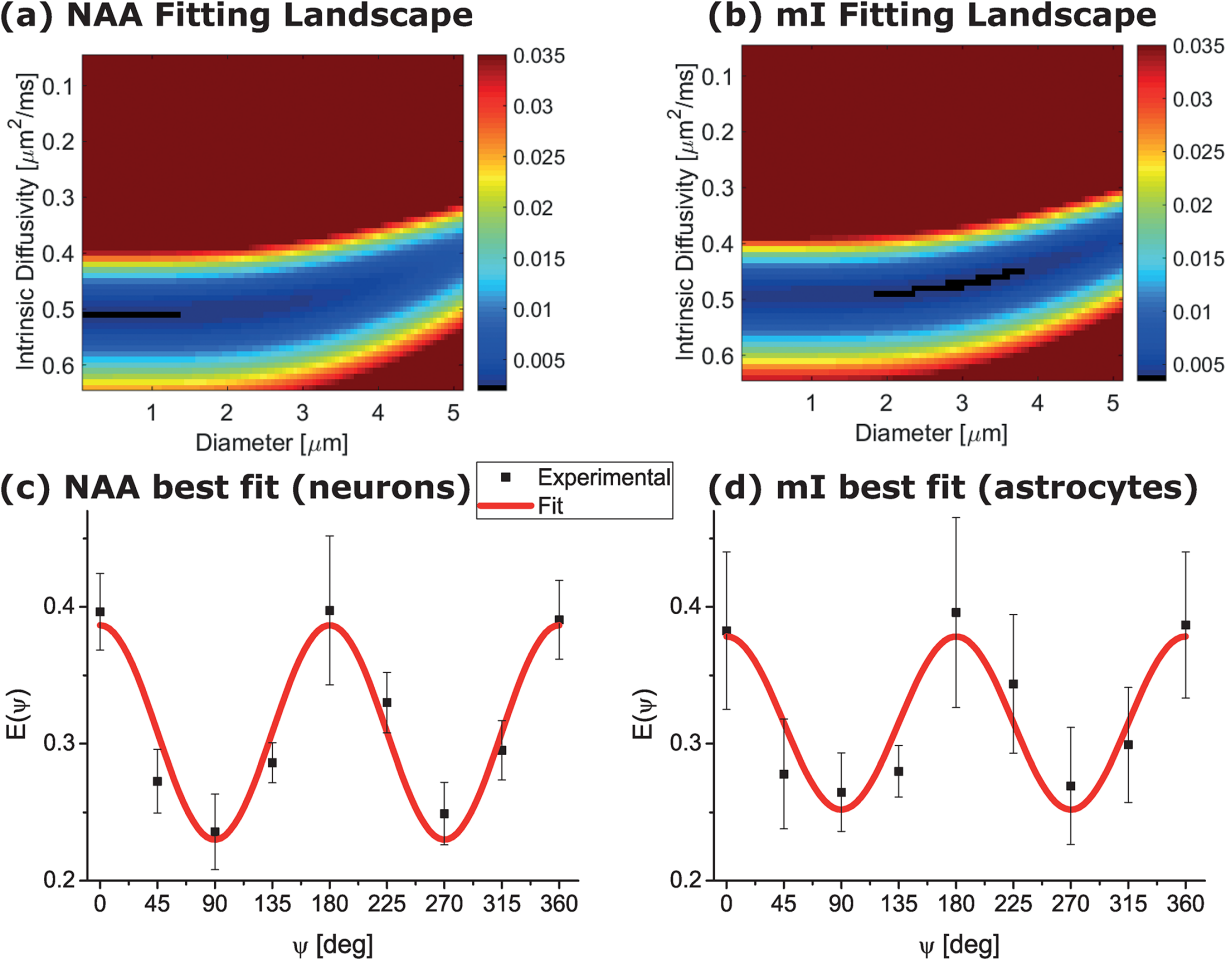
Fitting the experimental data to the randomly oriented infinite cylinder model. (a,b) Fitting landscape for NAA and mI, respectively, showing the residuals as function of different intrinsic diffusivity and diameter, respectively. (c,d) Plots of the best fit model parameters alongside the experimental data for NAA and mI, respectively. Notice that the astrocytic processes appear to be somewhat larger than the neurites.

Fig 8 analyzes the observed signal oscillations vis-à-vis models arising from the MISST toolbox. Given that NAA and mI are purely intracellular, this assessment suggested that infinite, randomly-oriented cylinders are the most appropriate model for interpreting the DDE data shown in Fig 7. Hence, the signals were fit to this model using two fitting variables: the intrinsic diffusivity and the diameter. Fig 8a and 8b show the residuals’ structure; i.e., how these fits vary with intrinsic diffusivity and cylinder diameter for both metabolites. For NAA, the minima suggest a highly eccentric pore, occurring below diameters of <1.5 μm (the absolute minimum was *d* = 0.1 *μm* and *D*_0_ = 0.51 *μm*^2^/*ms*); for mI, the diametric minima appear to be concentrated between ~2–4 μm (the absolute minimum for mI was *d* = 3.1*μm and D*_0_ = 0.47*μm*^2^/*ms*). While the aforementioned figures yielding the absolute best fits do not reach statistical significance, likely due to a small sample size, a trend is nevertheless observed (p<0.1). Importantly, notice that such MISST-based analyses take into account all actual DDE experimental parameters that were used, and in particular, the effects of finite gradient duration [78], and non-infinite diffusion times.

## Discussion and conclusion

The results described above demonstrate that ultrahigh field MRS enables one to target the morphologies of specific elements in the highly complex neural tissue architecture. Neurons and astrocytes are both highly heterogeneous populations, and in a (5 mm)^3^ voxel centered in rat brain, one can expect to find numerous types of neurons (glutamatergic, GABAergic and, due to the inclusion of basal ganglia within the location of our voxel, also dopaminergic and other modulatory neurons). These populations of neural cells are characterized by relatively large spherical or amorphous cell bodies, ranging between 5–30 μm. Similarly, the majority of mI-containing glial cells in the targeted region of the brain will be protoplasmic and fibrous astrocytes; cells comprised of slightly elongated somas, the majority of which are in the 10–30 μm range [79]. By contrast to these relatively large and weakly eccentric somatic structures, the neuronal neurites and the astrocytic processes exhibit a dramatically different morphology: their diameters are at least one order of magnitude smaller than the soma—in the 0.1–3 μm range—and exhibit a high prevalence of submicron diameters, while their lengths can reach hundreds of microns. Here, we used the mI and NAA signals to spectroscopically distinguish between the two cell types, and the DDE filter to impart additional microstructural sensitivity to the experiment. The strong DDE modulations that we observe, backed as they were by an infinite-cylinder best-fit model, impart on the signal of these metabolites evidence of constrained cellular morphologies. Although somewhat compounded by the slight macroscopic anisotropy that was found in SDE-based experiments (Fig 6), and although the DDE experiments here performed spanned the three orthogonal planes but were not performed in a fully rotationally invariant fashion due to time limitations, the absence of a clear phase in the DDE modulations suggests that very little residual ensemble anisotropy contributed to these signals’ modulations (c.f. Shemesh et al, 2012). Our findings then suggest similar—but not identical—radii for neurons and astrocytes probed by NAA and mI, respectively: <1.5 μm diameters are observed for the former and slightly larger diameters for the latter. In fact, the fit minima of <0.1 μm and ~3.1 μm for the NAA and mI compartments are in excellent agreement with the numbers obtained from Palombo et al’s q-dependent measurements (Palombo et al, 2016b), from which diameter dimensions of 0.04 μm and 3.1 μm were obtained for NAA and mI compartments, respectively. The intrinsic diffusivities extracted here are slightly larger than those calculated by Palombo *et al*’s model, likely reflecting subtle differences in the diffusion parameters with which the signals were encoded in both studies. Nevertheless, NAA’s and mI compartment values are in good agreement with the dimensions of randomly oriented neurites and astrocytic processes, suggesting that our measurements successfully targeted these randomly oriented, subcellular structures. The highly eccentric morphologies extracted therefore seem to reflect that NAA diffuses predominantly within the randomly oriented neurites, and mI diffuses mainly within the randomly oriented (star-shaped) astrocytic protrusions. It is worth pointing out that recent studies suggested that tCho and tCre are more concentrated in astrocytes than in neurons [52,54], and that a similar approach to the one taken here could be used to extract their eccentricities in a model based fashion [43]. Myo-inositol, however, could be a more specific marker, as both tCre and tCho are likely not as specific to astrocytes as mI, and may thus introduce partial volume effects onto the measurements.

The eccentric cell processes detected here by DDE could be important in the context of measuring *in vivo* brain plasticity [80], in psychiatric [81] or in the context of degenerative diseases [82]. It is worth noting that if desired, other specialized filters targeting other subcellular features (most notably, the spherical cell bodies), with minimal sensitivity towards the randomly oriented neurites, can be designed using existing diffusion-based frameworks [35,36,66–68]. These filters also could potentially be useful for modeling more complex aspects of the tissues under investigation, such as its underlying size distributions or, if rotationally invariant DDE acquisition schemes are employed [29,35], to disentangle macroscopic anisotropy from microscopic anisotropy. Even more sophisticated encodings of the diffusion gradients [83,84] could be envisioned, which would benefit from the RE MRS ultrahigh field operation.

Having demonstrated the feasibility of such experiments in a preclinical setting, it is worth reflecting on potential extensions of this approach to human studies. This can be carried out on the basis of SNR considerations, according to which SNR in MRI/S scales approximately linearly with voxel volume, as well as with field. High field human scanners typically operate at three times lower Larmor frequencies than the field used herein, ca. 7 T in human vs 21.1 T in animals. However, voxel volumes in typical human-oriented MRS studies are ca. 5–10 times larger than the (5 mm)^3^ volume used in this work. Hence, the onus of losing SNR via field reduction could be compensated by a larger voxel size. Because DDE measurements deliver information on randomly oriented structures, its use for investigating the underlying microstructure gray matter regions (which are typically larger than white matter structures), could be appealing. Moreover, because metabolites have relatively long T_2_ times but rather short T_1_s at decreasing fields [43,47,85], lower field studies could accommodate the longer excitation pulses characteristic of human scanners, covering similar bandwidths (in ppm) as used in this animal study, while suffering little penalties in terms of spectral selectivity. The application of long gradient pulses to compensate for the six times lower gradients available in clinic scanners would be a severe complicating factor in such human tests; however, the emergence of scanners with strong gradients (7–10 G/cm) for clinical applications, or of even with stronger gradients of the kind employed in the Human Connectome project (30 G/cm) [86] should permit for the observation of amplitude modulation that gives rise to DDE microstructural information—even if the sequence becomes somewhat more biased towards long T_2_ species. Diffusion MRS experiments at 7 T are already providing intriguing results [54,55,58]; extending these towards direct microstructural measurements by DDE or by other approaches for quantifying μA—such as qMAS [41] or isotropic/anisotropic encoding approaches [34]–promise additional interesting methodologies towards biomarking normal process such as neural plasticity associated with memory and learning [80], or neurological diseases that may impact cellular morphologies and subcellular features of neurons and astrocytes differentially [1–3]. It should be noted that the spin-echo and LASER modules used in this study could be quite SAR-intensive; still, the relatively low duty cycles used in these MRS determinations might offset such penalties.

In summary, this study presented an approach to interrogate specific cellular microstructures based on a double diffusion encoding approach that specifically targets metabolites that are mostly compartmentalized in different kinds of cells. The resonances of mI and NAA were chosen as cellular-specific targets, and they could be studied thanks to the high sensitivity and fidelity provided by ultrahigh field RE-MRS. A new DDE-CPMG sequence was used, which can in principle reduce considerably the cross-terms between internal and external gradients (Fig 4). Besides their usefulness in high field applications, where susceptibility and problematic shimming may be limiting factors, selective CPMG modules may also offer robustness against J-modulation for coupled metabolites. It was then observed that both metabolites can undergo restricted diffusion and exhibit microscopic anisotropy, showing trends towards slightly different eccentricities in neuronal and astrocytic cells over a cohort of *in vivo* tests. The DDE filter used was “monochromatic”, in the sense of involving a single q-value and a single diffusion time; other types of filters could be designed to yield sensitivity to other elements and morphologies in the cellular microstructure. DDE RE-MRS’s potential for reporting microstructural features of cellular and sub-cellular domains is thus promising, and could be harnessed for understanding healthy and degenerative processes within the neural tissues. This bodes well for a better understanding of brain’s cell biology and physiology in health and disease.

## Supporting information

S1 File The supporting information contains 262 folders with all the data employed in reaching the conclusions of this paper. This includes the DDE MRS data for every animal, sorted by gradient strength/direction and using the nomenclature animalX\3Y –where X was the X^th^ animal studied and Y was the Y^th^ experiment performed in that animal. The first experiment in every folder always corresponds to G = 0 while the next experiments follow the relative orientations psi = 0:45:360, (corresponding to experiments 31–39). If an experiment number has the suffix 'o' it means that an artifact was detected and that the experiment was repeated. Each folder contains the collected data plus acquisition information, in standard Bruker Paravision format. These sets include all the data needed to repeat the experiments and reach this manuscript’s conclusions.
(RAR)

## Abbreviations

CPMG: Carr-Purcell-Meiboom-Gill
DDE: Double Diffusion Encoding
LASER: Localization by Adiabatic SElective Refocusing
mI: myo-Inositol
MRI: Magnetic Resonance Imaging
MRS: Magnetic Resonance Spectroscopy
NA: Number of Averages
NAA: N-Acetylaspartate
RE MRS: Relaxation Enhanced Magnetic Resonance Spectroscopy
RF: radiofrequency
SDE: Single Diffusion Encoding
SNR: Signal to Noise Ratio
TE: Echo time
TR: Repetition time
μA: Microscopic anisotropy
